# Garlic essential oil alleviate oxidative stress, inflammation and microbiota dybiosis from small intestinal damage in lipopolysaccharide-challenged weaned piglets

**DOI:** 10.1186/s40813-025-00461-6

**Published:** 2025-09-30

**Authors:** Yun Chen, Wenjing Song, Chongbo Sun, Yao Chen, Xinyi Chen, Shuyan Chen, Qiuling Chen, Kai Wang

**Affiliations:** 1 College of Biology and Agriculture, Guangdong Provincial Key Laboratory of Utilization and Conservation of Food and Medicinal Resources in Northern Region, Shaoguan University, Shaoguan, 512005 Guangdong China; 2 Guangdong Provincial Key Laboratory of Agro-animal Genomics and Molecular Breeding, College of Animal Science, South China Agricultural University, Guangzhou, P.R. China; 3 Nanjing Agricultural University, Nanjing, 210095 China

**Keywords:** Piglet, Nutrition, LPS, Immunology, Diarrhea, Garlic essential oil

## Abstract

This study evaluated the effects of garlic essential oil (GO) supplementation on growth performance, immunity, antioxidant capacity, and small intestine health in weaned piglets. Piglets fed 1 g/kg GO exhibited significantly higher final body weight and average daily gain, coupled with reduced feed-to-gain ratio, indicating enhanced growth performance. In contrast, 0.5 g/kg GO showed no effect, while 1.5 g/kg negatively impacted feed intake and final weight. Immunologically, 1 g/kg GO reduced serum TNF-α and MDA levels, improved jejunal SOD activity, and enhanced antioxidant gene expression (*Sod1*, *Gpx1*). Morphologically, 1 g/kg GO increased jejunal villus height and villus-to-crypt ratio while upregulating tight-junction proteins (*Zo-1*, *Claudin-1*). After LPS challenge, GO reduced diarrhea incidence and decreased serum pro-inflammatory cytokines (IL-1β, TNF-α). GO also mitigated mitochondrial oxidative stress by lowering MDA and ROS levels while enhancing mitochondrial membrane potential in jejunum. However, residual ROS levels remained higher than in the control group. Additionally, GO suppressed LPS-induced NFκB expression while increasing IL-10 abundance, indicating improved gut inflammation in jejunum. Moreover, GO reshaped the LPS-induced microbial dysbiosis, significantly increasing the abundance of *Lactobacillus* while markedly reducing the abundance of *Escherichia coli.* Notably, although cefalexin is superior to GO in terms of anti-inflammatory effects, it may have a negative impact on gut microbiota homeostasis. These findings suggest that 1 g/kg GO optimally enhances growth performance, intestinal health, and immunity in weaned piglets while partially alleviating LPS-induced intestinal disruption.

**Supplementary Information:**

The online version contains supplementary material available at 10.1186/s40813-025-00461-6.

## Graphical abstract

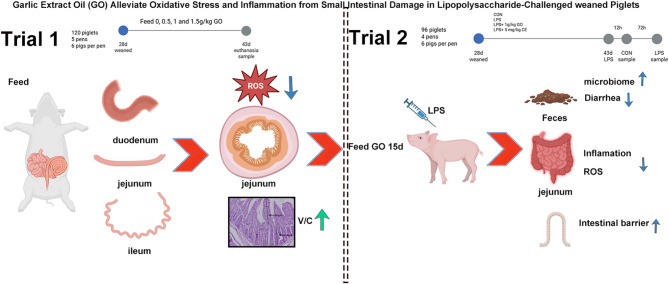

## Introduction

Post-weaning diarrhea is a common gastrointestinal disorder. This condition is marked by symptoms such as watery stools, decreased appetite, and stunted growth [1]. The incidence of post-weaning diarrhea varies among pig production systems and can have significant economic implications. Post-weaning diarrhea has detrimental effects on piglet health and growth. Diarrhea causes electrolyte imbalances, dehydration, and nutrient malabsorption, which leads to weight gain reduction, increased mortality rates, and immune function impairment. The economic consequences are reduced feed efficiency, prolonged time to market, and increased veterinary and medication costs.

Antibiotics in pig feed can promote growth and prevent disease, but they can also lead to increasing antibiotic resistance of bacteria and posing a potential risk to human health. In recent years, more and more countries have started to restrict or prohibit antibiotics usage in enhancing animal growth performance to reduce the risk of antibiotic resistance [2]. The U.S. has begun to strengthen its regulation of antibiotics in feed [3]. Alternative products have been widely researched and applied, including probiotics, prebiotics, acidifiers, plant extracts, and others [4]. These alternatives can improve the gut microbiota of pigs, enhance immunity, prevent disease, improve growth performance, and simultaneously avoid the risk of antibiotic resistance.

Garlic essential oil (GO), derived from garlic, is an organic compound possessing a diverse array of biological properties such as antioxidant, antibacterial, antiviral, hypotensive, and lipid-lowering effects [5]. Its incorporation into animal feed has garnered significant interest, given its potential to bolster the immune system and growth of livestock and poultry, while also preventing diseases. GO’s natural antimicrobial properties can mitigate the presence of harmful bacteria in feed, thereby lessening their impact on animals [6]. Furthermore, GO can enhance the immunity of livestock and poultry, thus preventing the occurrence of diseases. And GO can promote livestock and poultry growth performance and enhance muscle growth and feed utilization [7]. Although previous studies have demonstrated that GO can enhance the immune response in livestock and poultry, its effects on weaning stress in piglets and the potential mechanisms by which it regulates intestinal inflammation to prevent diarrhea remain unclear.

Endotoxins such as lipopolysaccharides are known to trigger immune reactions in the gut, sparking inflammation, compromising immune capabilities, and leading to deteriorated health in animals. Our study postulates that GO might fortify the intestinal barrier, which could lead to a reduction in gut inflammation and oxidative harm, a decrease in diarrhea frequency, and ultimately, improved growth in weaned piglets. To validate this hypothesis, we initiated by incorporating multiple levels of garlic essential into the diet to evaluate its effects on the growth performance, antioxidant capacity and immune competence of the piglets. We then proceeded to assess how GO compares to antibiotic intervention in an LPS-induced intestinal injury model in piglets, focusing on its potential to preserve intestinal barrier completeness and modulate oxidative stress and inflammatory responses.

## Materials and methods

This study consisted of two independent in vivo trials. Trial 1 was carried out from March 1 to March 16, 2023, and aimed to evaluate the effects of garlic essential oil (GO) supplementation on growth performance and intestinal health in weaned piglets. Trial 2 was conducted from April 1 to April 18, 2023, and focused on investigating the effects of GO on immune response, antioxidant capacity, and diarrhea incidence following an intraperitoneal lipopolysaccharide (LPS) challenge.

Both trials used healthy 28-day-old weaned piglets with similar initial body weights. All animals were housed in environmentally controlled nursery pens with slatted floors, provided with ad libitum access to feed and water. The room temperature was maintained at 28–30 °C during the entire trial period, and piglets were monitored daily for general health status. The animal experiment following the strict protocols outlined by the National Institutes of Health, ensuring the highest standards of animal care and experimental conduct. The study was granted approval by the Animal Welfare Committee at Shaoguan University (2021-11sc-001).

### Preparation of Garlic essential oil

In this study, we used a garlic essential oil ( purity ≥ 98%, HPLC-verified ) derived from garlic powder (Product Code: RB563 Hubei Runbo Pharmaceutical Co., Ltd) which was extracted through a standardized steam distillation and oil separation process performed. Approximately 15–20 kg of garlic powder is required to produce 1 kg of essential oil, depending on the moisture content and extraction yield.

In the preparation of the micro-encapsulated essential oils, a mixture comprising stearic acid and additional wall materials made up 65% of the formulation, while the garlic essential oil constituted the remaining 35%. This blend was heated to achieve a uniform paste-like texture. Subsequently, the mixture was transformed into fine droplets through a condensation spray process within a tower, which facilitated the rapid solidification into microcapsules. To further enhance the stability of the garlic essential oil, we incorporated composite mineral materials and emulsifiers into the mixture. The final step involved processing the formulation until a transparent, protective film encapsulated the microcapsules, resulting in a two-layer coating that shielded the active ingredients and ensured their stability throughout the experiment (Table S1).

### Animals, experimental design, and diets

All piglets were sourced from the same commercial farm and were of uniform breed background (Landrace × Yorkshire × Duroc). In total, the piglets originated from 18 different litters, and the litters were sired by five different boars to ensure genetic diversity. Weaned piglets were stratified by body weight and sex and then randomly assigned to four treatment groups using the RAND function in Microsoft Excel. Each treatment group consisted of five pens, with six piglets per pen (three males and three females). Piglets were not moved between pens throughout the study.

In the first experiment, growth performance and intestinal health of weaned pigs were investigated. One hundred and twenty, 28 days weaned piglets (Landrace × Yorkshire × Duroc, 6.12 ± 0.90 kg) were used in the study. The piglets were assigned randomly to four treatment groups according to their body weight and gender, each consisting of five pens and six pigs per pen, three males and three females. control group (received the basal diet), 0.5 GO group (basal diet + 0.5 g/kg GO, garlic essential oil per kg of feed), 1 GO group (basal diet + 1 g/kg GO), and 1.5 GO group (basal diet + 1.5 g/kg GO). All diets provided met or exceeded the nutrient requirements recommended by the National Research Council (NRC, 2012), as shown in Table S2. The piglets were allowed ad libitum access to their diets for 15 days. Throughout the study period, all piglets were provided unrestricted access to both food and water. The occurrence of diarrhea among piglets in each pen was recorded. Based on preliminary observations, piglets tended to eat in clusters around routine feeding times, particularly in the morning, midday, and evening. Therefore, we conducted fecal observations at fixed time periods — approximately two hours after the typical peak feeding periods — to ensure consistency in monitoring and to capture the most representative defecation activity. The diarrhea index scoring system was as follows: 0 denoted normal feces with solid consistency, 1 indicated moist feces with semi-solid consistency, 2 represented mild diarrhea with loose feces, and 3 denoted severe diarrheas with watery feces. Both mild and severe diarrhea instances were classified as diarrhea. The diarrhea incidence was calculated by normalizing the number of weaned piglets experiencing diarrhea to the total number of weaned piglets per pen.

In the second experiment, immune response, antioxidant capacity and diarrhea incidence post-LPS challenge were investigated. Ninety six 28 days weaned piglets randomly assigned to four groups: a control group, LPS-treated groups (injection on the 15th day), LPS + 1 g/kg garlic essential oil in feed (LPS + GO, lasting for 18 days), and LPS + 5 mg/kg cefalexn in feed (LPS + CE, medication on the 15th day, lasting for 3 days), 4 pens, 6 piglets per pen, three males and three females. The age of the piglets and the duration of feeding were consistent with those in the first experiment. Piglets in the LPS-treated groups were intraperitoneally injected with Escherichia coli LPS (Escherichia coli serotype 055: B5, Sigma Chemical Inc., St. Louis, MO, USA) at a dosage of 120 µg/kg body weight (BW), and then euthanized 72 h after the LPS challenge. Piglets in the control group were euthanized 12 h after injection with an equivalent volume of sterile saline. Piglets in the control group were euthanized 12 h after saline injection to represent the physiological baseline. In contrast, piglets in the LPS-challenged groups were euthanized 72 h after LPS injection to allow sufficient time for the development of systemic inflammatory responses and to evaluate the potential therapeutic effects of GO and CE. The timing difference was designed to best capture both healthy baseline conditions and the full inflammatory progression post-challenge. Cefalexn was supplied by Shanghai Dongyitang Biotechnology Co., Ltd. China (CAS:15686-71-2, DYT013, HPLC > 99%). All fecal samples from pigs in both Experiment 1 and Experiment 2 were tested for the presence of enterotoxigenic *Escherichia coli* pathogens.

### Data collection

Preceding the morning feeding, 5 ml blood samples were collected from random three pigs per pen, totaling 15 samples per group. The collection was performed via puncture of the anterior vena cava. Post-collection, the blood samples were subjected to centrifugation at 3000 ×g for a duration of 15 min at a temperature of 4 ℃ to isolate the serum, which was then cryopreserved at -80 ℃. After the blood sampling, two pigs from each pen underwent anesthesia using an injectable sodium pentobarbital solution at a dosage of 50 mg per kilogram of body weight, followed by euthanasia through exsanguination. The small intestine was then carefully removed, and 4-centimeter segments of the duodenum, ileum and jejunum were extracted. To ensure comparability, these segments were obtained from consistent locations along the length of the duodenum, ileum and jejunum. The harvested tissues were briefly washed with a 0.1 M phosphate-buffered saline solution at a pH of 7.2 prior to fixation in a 10% formaldehyde-phosphate buffer for future histological and immunohistochemical assessments. Additionally, about 3 g of the jejunal mucosa were quickly frozen in liquid nitrogen and kept at -80 ℃ for the subsequent extraction of total RNA and proteins. In both experiments, fecal samples were collected directly from the rectum of each piglet using sterile disposable gloves at the end of the trial period, prior to euthanasia. Samples were immediately placed into sterile tubes, snap-frozen in liquid nitrogen, and stored at − 80 °C until analysis. For pigs in the LPS-challenged groups, sampling was conducted 72 h after LPS injection, while control piglets were sampled 12 h after saline injection, consistent with their respective euthanasia time points. For molecular analyses such as serum cytokines and oxidative stress markers, blood samples were originally collected from 15 piglets per treatment group (3 piglets per pen from 5 pens). Due to budget constraints, a subset of these samples was analyzed: two sample was randomly selected from each pen, resulting in *n* = 10 for that group. Each sampled piglet was treated as an individual experimental unit for the statistical analysis of molecular data.

### Elisa assay analyses

The concentration of Tumor Necrosis Factor-alpha (TNF-α), and Interleukin-1beta (IL-1β) in the serum samples were quantified using commercially available ELISA kits (beyotime, china). Briefly, the ELISA plates were coated with specific antibodies against TNF-α, IL-1β and incubated overnight at 4 °C. After blocking the plates with blocking buffer, the serum samples and standard solutions with known concentrations of the respective cytokines were added to the wells and incubated for a designated period. After washing the plates to remove unbound substances, specific detection antibodies were added and incubated. Following a secondary incubation with an enzyme-conjugated secondary antibody, a substrate solution was added, and the plates were incubated in the dark for a specified time. The reaction was stopped using a stop solution, and each well’s optical density was measured at the appropriate wavelength using a microplate reader.

### Oxidative stress detection

The oxidative stress markers in the intestinal segments of the piglets by measuring the superoxide Dismutase (SOD), malondialdehyde (MDA), Catalase (CAT) and glutathione peroxidase (GSH-Px) in the duodenum, jejunum, and ileum were assessed. These assays were conducted using spectrophotometric analysis kits procured from Jiancheng Bioengineering Institute in Nanjing, China, following the protocols provided by the manufacturer.

### Morphological analysis

The jejunum samples, once fixed, were removed from their fixative and advanced through a series of ethanol and chloroform solutions to achieve dehydration. The dehydrated tissues were embedded in paraffin, from which microtome-cut cross-sections were prepared. These sections were then subjected to staining with hematoxylin and eosin. During morphometric assessment, a pair of representative sections from each jejunum sample was affixed to individual slides, and a total of ten well-defined and properly aligned crypt-villus complexes were selected at random for detailed measurement. The height of the villi (VH) was determined by measuring the distance from the peak of each villus to the base of the adjacent villi, whereas the depth of the crypts (CD) was charted from the base of the villi trough to the level of the basal membrane. The morphometric assessments were performed utilizing an Olympus BX51 microscope, which was fitted with a DP70 digital camera and JD801 morphologic image analysis software, both from Olympus Corporation in Tokyo, Japan.

### Quantitative real-time polymerase chain reaction (gPCR)analyses

We extracted total RNA from the intestinal tissue samples utilizing the TRIzol reagent Invitrogen, Carlsbad, CA, USA) in accordance with the provider’s guidelines. RNA purity and concentration were assessed using the Thermo ND2000 instrument (Thermo, CA, USA), with an acceptable A260/A280 ratio of 1.8–2.1 and an A260/A230 ratio above 2.0.RNA integrity was confirmed by 1% agarose gel electrophoresis.Subsequently,1 ug of high-quality total RNA was reverse transcribed into complementary DNA (cDNA)using the Superscript III enzyme (Invitrogen), following the manufacturer’s protocol. The expression levels of inflammation-related genes were evaluated through quantitative qPCR using SYBR Green dye (Thermo Fisher, Waltham, MA, USA). Amplification reactions were performed on the CFX Opus 384 Real-Time PCR Detection System (BioRad, Hercules, CA, USA). Each reaction mixture(20 uL) contained 2 uL of cDNA template,10 uL of SYBR Green Master Mix,0.5 uL each of forward and reverse primers (10 μm), and 7 uL of nuclease-free water. The amplification protocol consisted of an initial denaturation step at 95 C for 2 min, followed by 40 cycles of denaturation at 95 C for 10 s, annealing at 60℃ for 10 s, and extension at 72℃ for 30 s. Melting curve analysis was conducted to confirm product specificity and to rule out primer dimers. Each qPCR assay included six biological replicates and three technical replicates to ensure reliability and reproducibility. Negative controls (no template controls) were included for each primer set to detect potential contamination. The relative mRNA expression levels of target genes were calculated using the 2-^△△CT^ method, with β-actin as the endogenous control gene. β-actin stability under the experimental conditions was verified through preliminary tests to ensure its suitability as an internal reference. The primer sequences used for each gene and their amplification efficiencies (90–110%) are detailed in Supplementary Table S3. For microbial DNA extraction from fecal samples, the QIAamp-DNA stool mini kit (Qiagen, Hilden, Germany) was employed in strict adherence to the manufacturer’s instructions. DNA purity and concentration were measured using the Thermol ND2000 instrument, ensuring A260/A280 and A260/A230 ratios within acceptable ranges. The extracted DNA was stored at -80 ℃ until further analysis.

### Extraction of mitochondria from intestinal mucosa

Mitochondria were following a procedure outlined in prior research [8]. Started by homogenizing around 0.5 g of jejunal mucosa in a chilled MSH buffer, which is made up of 10 mmol/L HEPES, 200 mmol/L mannitol, 70 mmol/L sucrose, 1.0 mmol/L EGTA, and 2.0 mg/mL of serum albumin. Post-homogenization, the mixture was centrifuged at 1000×g for a duration of 10 min at a temperature of 4 °C. The supernatant obtained was then centrifuged again at a higher speed of 3500×g for another 10-minute interval at 4 °C to precipitate the mitochondrial pellet. The protein content of the extracted mitochondria was determined by performing the BCA assay [9]. The isolated intestinal mucosal mitochondria were then treated with 2 µmol/L 2’,7’-dichlorofluorescein diacetate, a compound capable of passing through the outer mitochondrial membrane. Post a 20-minute incubation period at ambient temperature, the fluorescence intensity was recorded using a fluorescence microplate reader [10]. To assess the mitochondrial membrane potential (ΔΨm), we employed a JC-1 ΔΨm detection kit from Beyotime Institute of Biotechnology in Haimen, China, adhering to the manufacturer’s instructions [11]. The mitochondria, once isolated, were resuspended in 0.5 mL of medium that contained 5 mmol/L JC-1, and the fluorescence levels were promptly determined with an FLx800 fluorescence microplate reader procured from BioTek in Winooski, VT, USA. The aggregation of JC-1 within the mitochondrial matrix varies with membrane potential; high potential leads to aggregation and red fluorescence emission, whereas low potential results in dispersed green fluorescence. Consequently, the ΔΨm of the intestinal mitochondria was calculated based on the ratio of red to green fluorescence intensity.

### Western blot

Proteins extracted from the intestinal tissues of the experimental samples were isolated using a standard lysis reagent (catalog #P0013B, Beyotime Biotechnology, China), enhanced with both a protease inhibitor (catalog #P1048) and a phosphatase inhibitor (catalog #P1082), all sourced from Beyotime, China. After the extraction process, the protein samples, now mixed with an appropriate amount of 1× loading buffer, were heated to achieve denaturation at a temperature of 95 °C for a duration of 10 min. The denatured proteins were then loaded onto either a 10% or 12% SDS-polyacrylamide gel for electrophoresis and later blotted onto polyvinylidene difluoride (PVDF) membranes. The membranes were incubated at room temperature for 1 to 2 h with a blocking solution composed of 5% BSA in Tris-buffered saline with Tween 20 (TBST). Following the blocking step, the membranes were incubated with primary antibodies specific to the proteins of interest—NFκB (product #10745-1-AP, Proteintech, Wuhan, China), and IL-10 (product #HZ-1145, Proteintech, Wuhan, China)—at 4 °C overnight in a light-protected environment. The next day, the PVDF membranes were thoroughly washed twice with TBST before the addition of a secondary antibody, which was sourced from Beijing Ray Antibody Biotech, China, and incubated for 1 to 2 h. The detection of protein bands was accomplished using an enhanced chemiluminescent substrate (ECL, catalog #P0018AM, Beyotime Biotechnology, China), and the signals were visualized and captured with the ChemiDoc MP imaging system (BIORAD), which was equipped with Image Lab 5.2.1 software. The subsequent data analysis was conducted utilizing ImageJ software (National Institutes of Health, Java 1.8.0).

### Fecal microbial community analysis

Fecal samples were thawed on ice prior to DNA extraction. A 0.1 g portion of each sample was weighed, and genomic DNA was isolated using the PowerSoil DNA Isolation Kit (MO BIO, USA) following the manufacturer’s protocol. To ensure high-quality DNA, purification was performed with the Mobio PowerClean DNA Clean-Up Kit, and the integrity of the extracted DNA was assessed through 1% agarose gel electrophoresis. For amplicon sequencing, the V1-V3 hypervariable region of the 16S rRNA gene was targeted using primers with unique barcode sequences at the 5’ end. Each sample was assigned an exclusive barcode for differentiation. The primers used were 27 F (AGAGTTTGATCCTGGCTCAG) and 533R (TTACCGCGGCTGCTGGCA). PCR amplification was carried out using TransGen AP221-O2 TransStart Fastpfu DNA Polymerase in a 20 µL reaction mixture containing 10 ng of DNA template, 0.4 µL of each primer (5 µM), 2 µL of dNTPs (2.5 mM), 0.4 µL of FastPfu Polymerase, and 4 µL of 5× FastPfu Buffer. The PCR conditions included an initial denaturation at 95 °C for 2 min, followed by 25 cycles of 95 °C for 30 s, 55 °C for 30 s, and 72 °C for 30 s, with a final elongation step at 72 °C for 5 min. Post-amplification, the PCR products were separated on a 2% agarose gel, and target bands were excised and purified using the AxyPrep DNA Gel Recovery Kit (AXYGEN, USA), with DNA eluted in Tris-HCl. Quantification of the purified amplicons was performed using the QuantiFluor™-ST Blue Fluorescence Quantification System (Promega, USA). Based on quantification results, amplicons from different samples were pooled in appropriate ratios to meet sequencing requirements. The prepared library was subjected to high-throughput sequencing on the Roche 454 platform.

### Statistical analysis

Prior to initiating the data analysis, we first confirmed that the dataset met the assumptions of normality and equal variances. The statistical processing of the data was performed utilizing the SPSS software, version 20.0. To discern differences among the four experimental groups, a one-way analysis of variance (ANOVA) was selected as the primary analytical approach. Whenever the ANOVA indicated a significant effect, we proceeded to conduct post-hoc comparisons using Tukey’s Honestly Significant Difference (HSD) test to pinpoint which specific groups differed from one another. The outcomes of the statistical tests are presented as the mean values accompanied by their standard error of the mean (SEM), with the threshold for statistical significance predefined at *p* < 0.05.

## Results

### Trial 1: Performance of weaned piglets

Piglets fed a daily ration containing 1 g/kg GO exhibited significantly higher final weight, and ADG (*p* < 0.05), along with a significant reduction in F/G (Table 1). These results indicate an improved growth rate in piglets when supplemented with GO. Conversely, piglets fed a daily ration containing 0.5 g/kg GO showed no differences in growth performance than the control group. Additionally, feed intake and final weight was significantly reduced with increasing GO to 1.5 g/kg concentration (*p* < 0.05).

**Table 1:** Effect of dietary Garlic essential levels on growth performance of piglets

Item	garlic essential oil (g/kg)				SEM	*P*-value
	con	0.5	1	1.5		
Initial weight (kg)	8.17	8.22	8.19	8.24	0.090	0.940
Final weight (kg)	13.17^a^	13.77^a^	14.96^b^	11.96^a^	0.162	0.048
ADG (g/day)	293.23^a^	312.16^a^	321.82^b^	248.20^c^	14.174	0.046
ADFI (g/day)	442.00^a^	449.98^a^	461.24^a^	406.15^b^	13.283	0.039
F/G	1.52^a^	1.44^b^	1.43^b^	1.63^c^	0.041	0.032

^abc^ means that different letters within a row indicate a significant difference (*p* < 0.05) between them. group: the control (basal diet), 0.5 group: basal diet + 0.5 g/kg garlic essential, 1.0 group: basal diet + 1 g/kg garlic essential, 1.5 group: basal diet + 1.5 g/kg garlic essential. SEM, total standard error of means (*n* = 30). ADG = average daily gain. ADFI = average daily feed intake. F/G = feed/gain ratio

### Trial 1: The serum immunity and antioxidant capacity of piglets

**Fig. 1:**
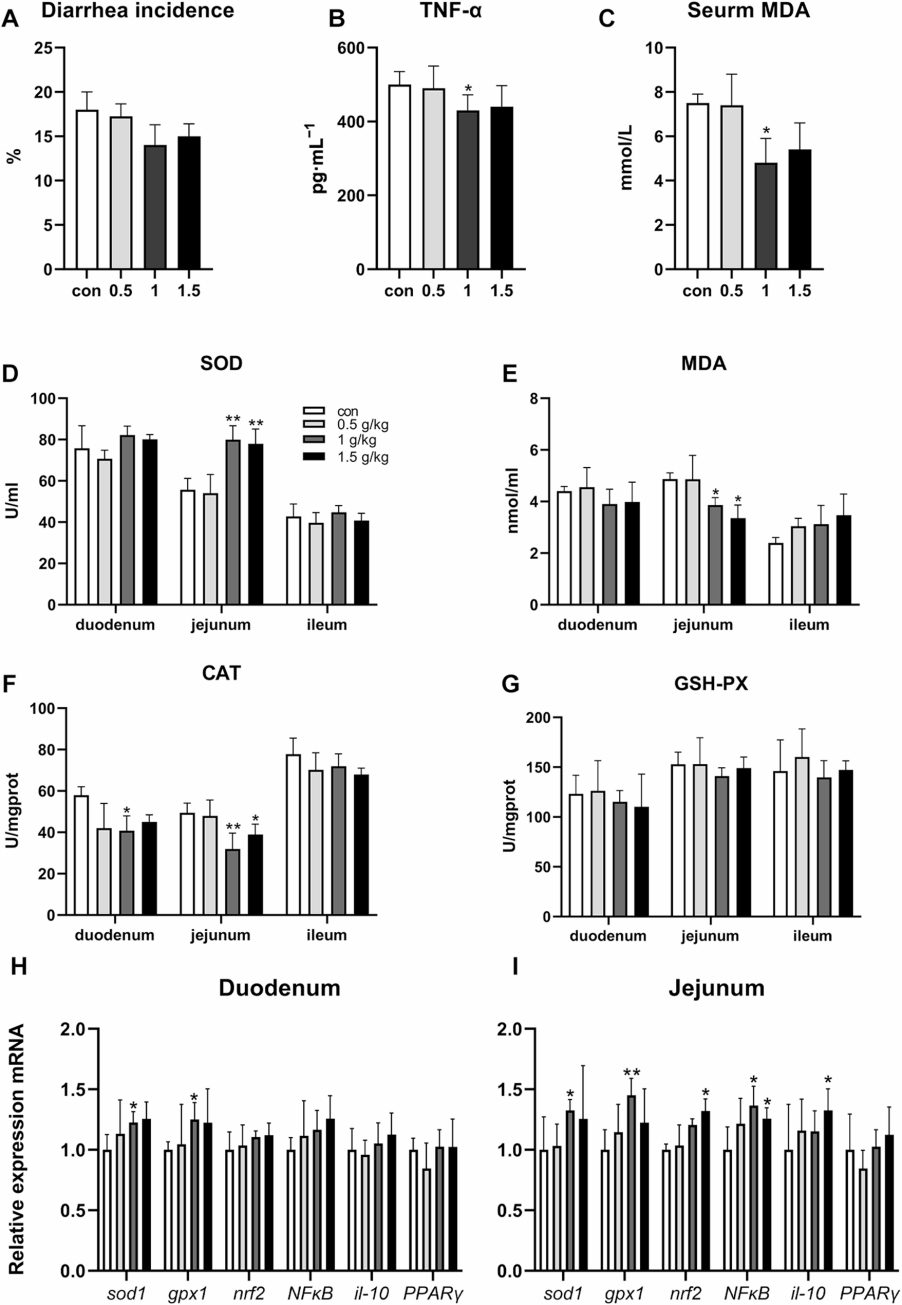
The effect of garlic essential oil on antioxidant capacity of piglets. (**A**) The incidence of diarrhea in piglets. (**B**) Serum TNF-α level. (**C**) Serum MDA level. (**D**) Different segments of the small intestine SOD level. (**E**) Different segments of the small intestine MDA level. (**F**) Different segments of the small intestine CAT level. (**G**) Different segments of the small intestine GSH-PX level. (**H**) Expression of antioxidant genes and inflammatory genes in the duodenum. (**I**) Expression of antioxidant genes and inflammatory genes in the jejunum. *n* = 10 *represents a significant difference *p* < 0.05. ** presents differ significantly (*p* < 0.01)

As the GO dosage in the feed rose, there was a corresponding decreased in the frequency of diarrhea among the piglets, as depicted in Fig. 1A. At dietary concentrations of 1 g/kg GO, the levels of the serum TNF-α and MDA were significantly diminished (Fig. 1B, C, *p* < 0.05). Moreover, at a 1 and 1.5 g/kg GO supplementation, there was a significant enhancement in the jejunum SOD activities, coupled with a significant decrease in MDA and CAT levels compared to the control group (Fig. 1D-F, *p* < 0.05). In addition, *sod1* and *gpx1* were significantly expressed in both the duodenum and jejunum, while *NFκB*, *nrf2*, and *il-10* were only significantly expressed in the jejunum (Fig. 1H, I, *p* < 0.05).

### Trial 1: The jejunum morphology health of piglets

**Fig. 2:**
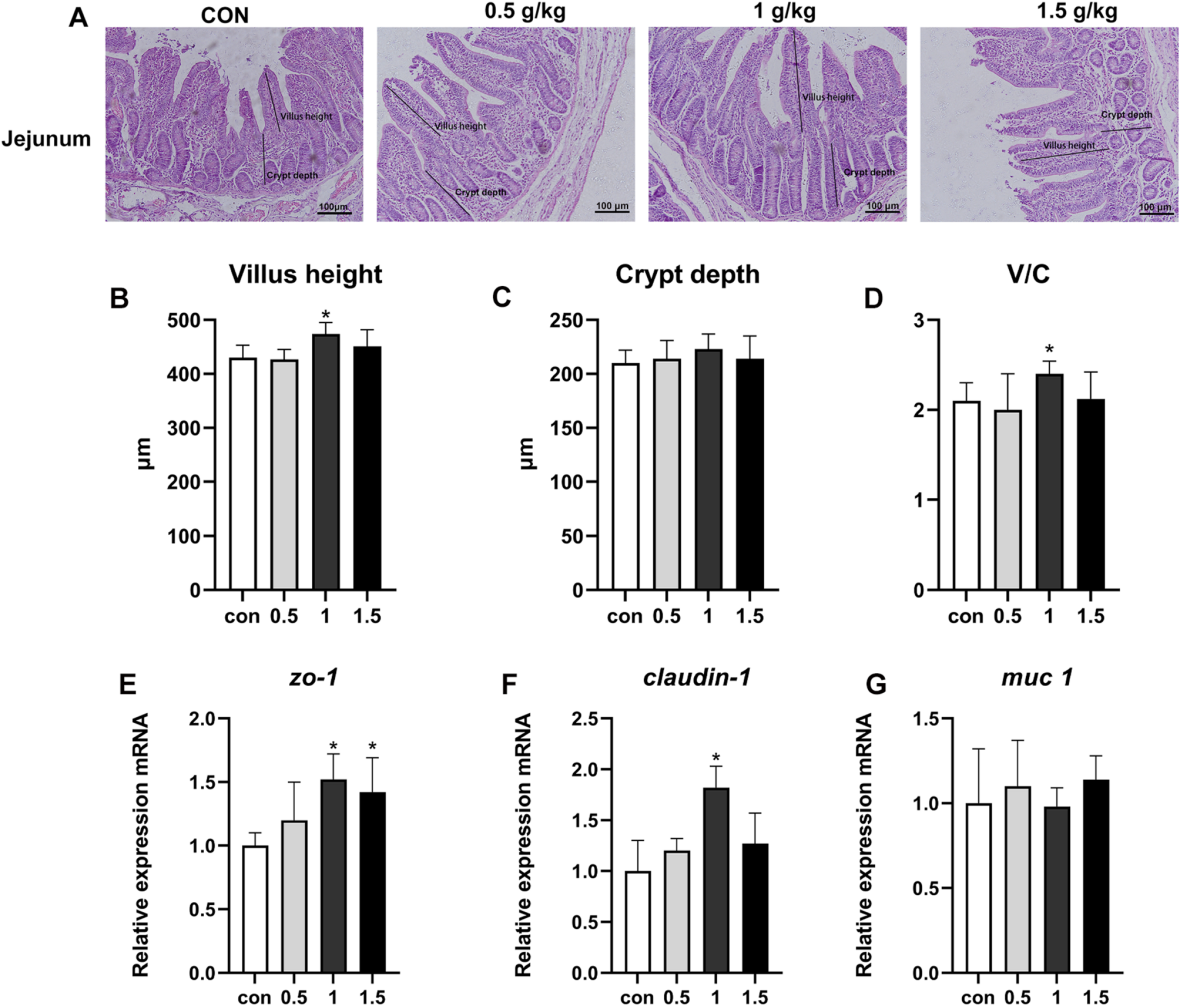
The effect of garlic essential oil on morphological jejunum of piglets. (**A**) Intestinal morphology of jejunum, black lines indicate the villi height and crypts depth, 100 μm; Con, basal diet; 0.5 basal diet + 0.5 g/kg garlic essential; 1.0, basal diet + 1.0 g/kg garlic essential; 1.5, basal diet + 1.5 g/kg garlic essential; (**B**) Villus height, (**C**) Crypt depth, (**D**) V/C villus height to crypt depth ratio; (**E-G**) mRNA expression of intestinal barrier proteins *Zo-1*, *Claudin-1* and *Muc1* (*n* = 10). *presents differ significantly (*p* < 0.05)

No significant variations in crypt depth were observed in the jejunum across the various groups treated with GO (Fig. 2C). In contrast, the group administered a diet containing 1 g/kg GO demonstrated a marked increase in both Villus height and V/C within the jejunum when compared to the control group, achieving statistical significance (Fig. 2B, D, *p* < 0.05). Furthermore, supplementation with 1 g/kg GO led to a significant upregulation in the mRNA levels of the tight junction proteins *zo-1* and *Claudin-1* (Fig. 2E, F, *p* < 0.05), while there was no substantial impact on the expression levels of *muc1* within the jejunum.

### Trial 2: GO alleviates LPS-induced diarrhea and immune disruption in piglets

**Fig. 3:**
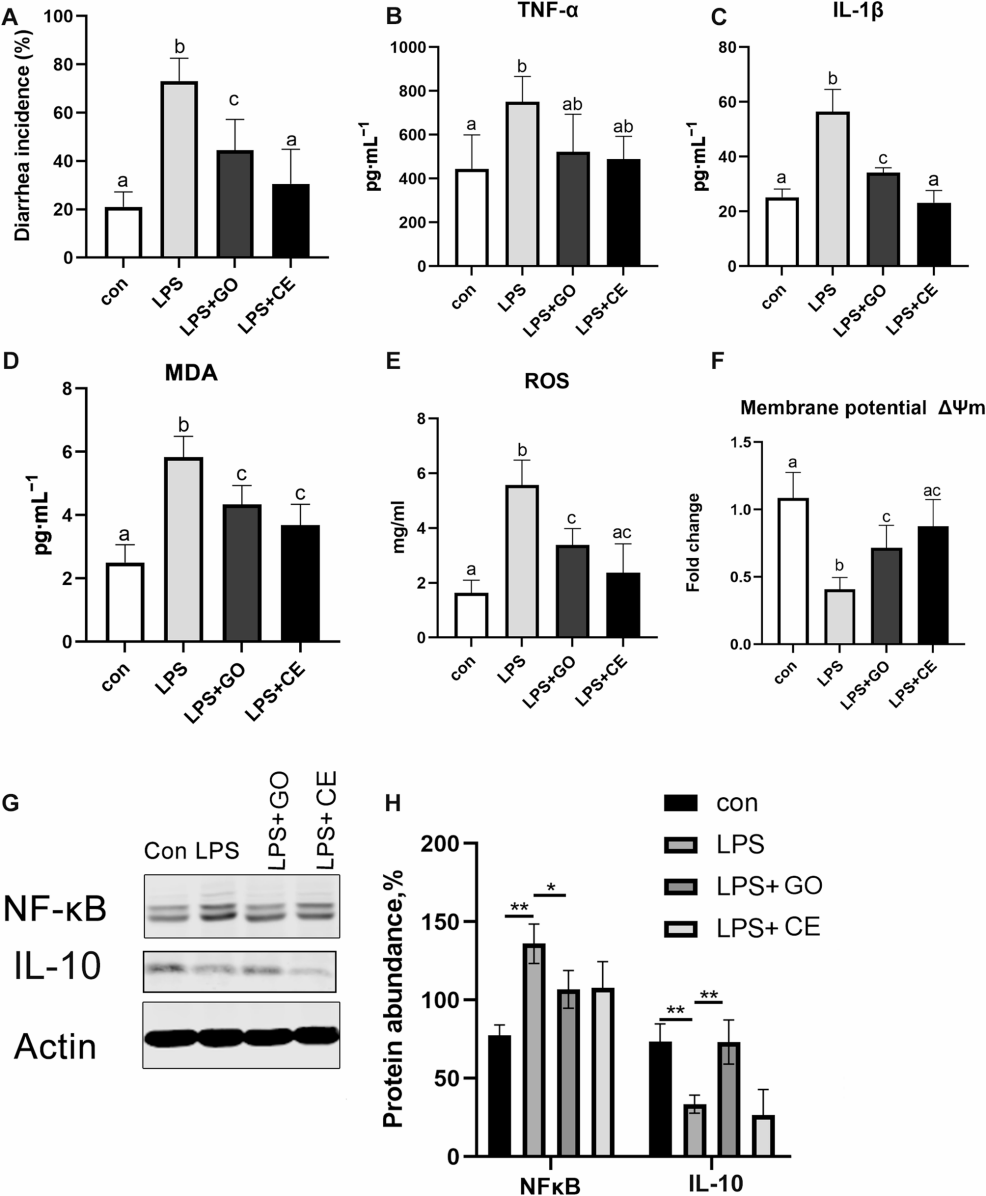
The effect of garlic essential oil on gut immunity of piglets attacked by LPS. (**A**) The incidence of diarrhea in piglets. (**B**) Serum TNF-α level. (**C**) Serum IL-1β. (**D**) Serum MDA level. (**E-F**) Mitochondrial ROS Levels and Membrane Potential in the Jejunum (**G**) Intestinal NFκB, and IL-10 protein levels. *n* = 8. different letters to indicate significant differences between groups (*p* < 0.05). Same or overlapping letters indicate no significant differences between groups. * presents differ significantly (*p* < 0.05). ** presents differ significantly (*p* < 0.01). GO: garlic essential; CE: cefalexn

Post the LPS challenge, as depicted in Fig. 3, there was a significant increase in diarrhea cases among the piglets (Fig. 3A). This was accompanied by an increase in serum levels of the pro-inflammatory cytokines IL-1β and TNF-α, which was statistically significant (Fig. 3B, C, *p* < 0.05). The GO treatment also led to an decrease in MDA and ROS in the jejunal mitochondria and a increase in mitochondrial membrane potential, both of which were significant (Fig. 3D-F, *p* < 0.05). Notably, the ROS levels in the LPS + GO group were still significantly higher than in the control group (*p* < 0.05), indicating that GO could not fully counteract the negative effects of LPS. GO also significantly reduced the increase in NFκB protein levels triggered by LPS, and increased abundance of IL-10 (Fig. 3G, H *p* < 0.05). In contrast, cefalexn, used as a comparative therapeutic control, showed superior efficacy in diarrhea to GO in managing LPS-induced effects.

### Trial 2: GO alleviates LPS-induced microbiota dybiosis in piglets

**Fig. 4:**
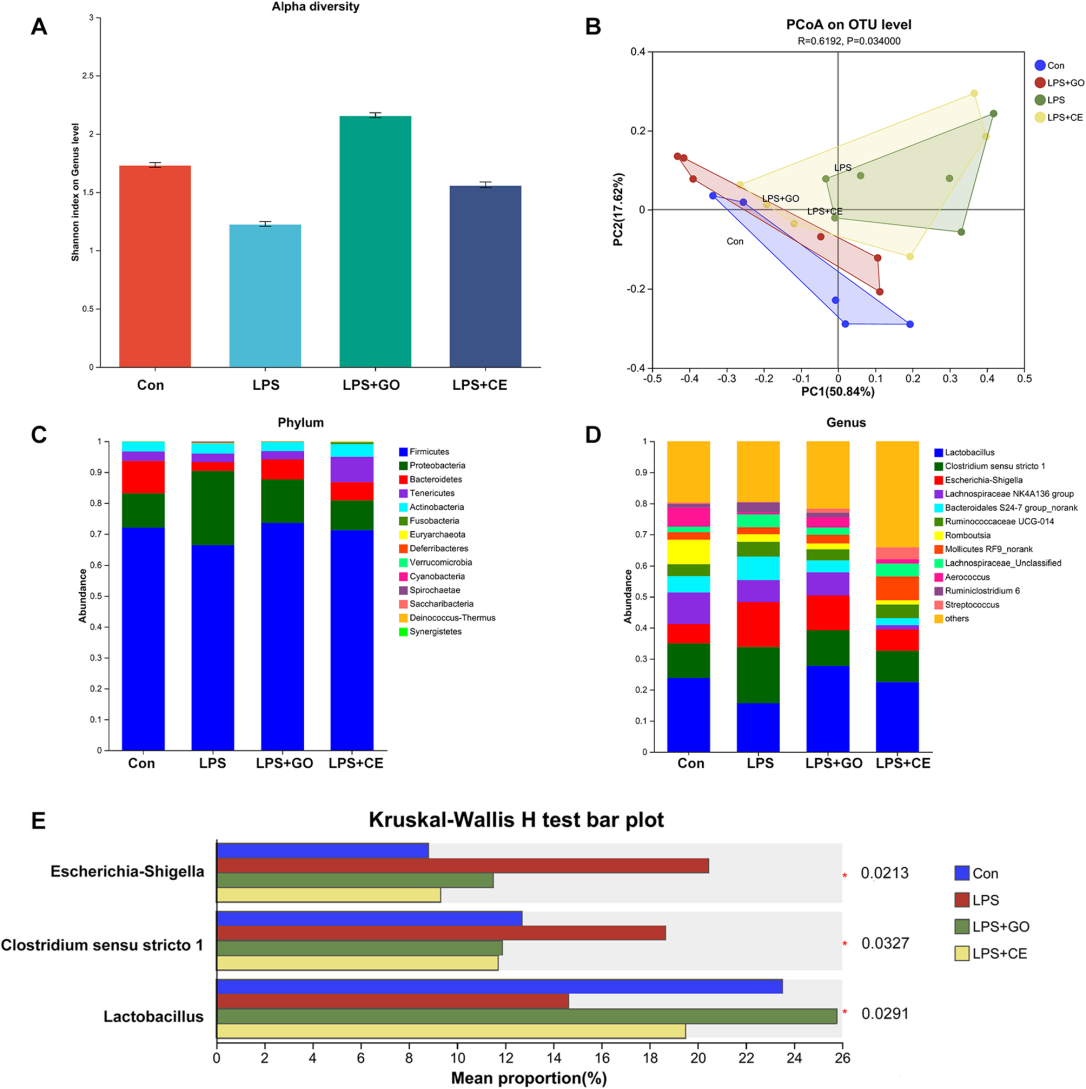
The effect of garlic essential oil on microbiota of piglets attacked by LPS. (**A**) α-diversity. (**B**) β-diversity. (**C**) Relative abundance in phylum. (**D**) Relative abundance in genus. (**E**) Significantly altered bacterial taxa. * presents differ significantly (*p* < 0.05). GO: garlic essential; CE: cefalexn

16 S rRNA sequencing analysis of fecal microbiota revealed significant alterations in bacterial composition among different groups. α and β diversity analysis demonstrated distinct clustering patterns among groups, suggesting that microbial composition was significantly affected by LPS (Fig. 4A,B) At the phylum level, Firmicutes and Bacteroidetes remained the dominant taxa across all samples, however in the LPS-treated group, the relative abundance of Proteobacteria was increased compared to the control group, indicating a potential microbial dysbiosis associated with inflammatory responses (Fig. 4C). At the genus level, several bacterial taxa exhibited significant differences in abundance (Fig. 4D). *Lactobacillus* was significantly enriched in LPS + GO compared to LPS (*p* < 0.05). In contrast, the relative abundance of *Escherichia-Shigella* was significantly reduced in LPS + GO (*p* < 0.05), implying a suppression of opportunistic pathogens (Fig. 4).

## Discussion

In previous studies, the addition of garlic essential to the diet has been shown to significantly enhance broiler chicken body weight and feed conversion ratio [12]. Moreover, feeding garlic essential to piglets resulted in a significant increase in body weight, although the duration of the trials was relatively short [13]. A study reported enhanced weight gain but no effect on post-weaning diarrhea using garlic powder [14], whereas our microencapsulated garlic extract oil significantly reduced diarrhea incidence, suggesting improved bioavailability and efficacy. Fermented garlic powder enhanced growth and reduced fecal *Escherichia-Shigella*, consistent with our findings of improved intestinal health [15]. Another study demonstrated dose-dependent growth benefits with garlic powder at higher inclusion rates [16]. In this experiment, including 0.5 g/kg of GO in the post-weaning diet did not affect the growth performance. In comparison, 1 g/kg of GO significantly improved the growth rate and reduced the feed-to-weight gain ratio. This suggests that an appropriate concentration of GO may possess properties as a digestive enhancer, thereby improving growth performance. Interestingly, further increasing the concentration of GO did not enhance the growth rate of piglets. We found that piglets in the 1.5 g/kg concentration group exhibited a significant decrease in daily weight gain, possibly due to the excessively high concentration of GO, which led to a spicy taste and odor, causing a significant reduction in ADFI and inadequate nutrient intake, resulting in delayed growth. Previous research findings have suggested that supplementation with GO can enhance the growth of pigs and other animals [13, 17, 18]. Nevertheless, certain research has indicated that incorporating garlic powder into the diet does not influence the growth performance of pigs being fattened for market [19]. This lack of effect might be due to the more mature immune system in these pigs, which offers robust resistance to diseases and consequently leads to fewer cases of diarrhea. Conversely, weaned piglets, with their less developed immune systems, are more susceptible to diseases and experience higher rates of diarrhea. Moreover, the efficacy of garlic powder could be influenced by the concentration and purity of its active component. Our findings indicated that GO can notably improve growth performance, decrease the frequency of diarrhea. Although the highest dosage of GO at 1.5 g/kg showed significant anti-inflammatory and antioxidant properties, its negative effect on the growth performance of piglets led us to exclude it from our recommendations for further consideration.

Weaning stress in piglets disrupts small intestine immune homeostasis, leading to increased intestinal permeability and inflammation. This stress response compromises the intestinal barrier, making piglets more susceptible to infections and diseases. The overexpression of pro-inflammatory cytokines such as IL-1β, TNF-α, and NFκB induces mucosal damage, alters epithelial permeability, and initiates inflammatory responses. Comparable anti-inflammatory effects of garlic have been reported in other mammalian models. For instance, aqueous garlic extract reduced TNF-α and oxidative stress in the kidneys of diabetic rats [20], and garlic supplementation in humans and rodents has been shown to decrease MDA and increase SOD levels [21]. A 1 g/kg GO significantly reduces the expression of pro-inflammatory cytokines IL-1β and TNF-α. Intestinal oxidative stress may contribute to structural damage in the gut. It has been demonstrated that the anti-oxidative stress of garlic is a potential factor that enhances the immune response. Our results indicate that, compared to the control group, 1 g/kg GO significantly reduced serum MDA concentrations and increased the activities of SOD. MDA is a byproduct of lipid peroxidation and serves as an indicator of oxidative stress. SOD is a critical antioxidants and free radical scavengers in the body. This study demonstrates that supplementation with 1 g/kg GO significantly alleviated weaning-induced oxidative stress, the antioxidant capacity of the jejunum is significantly enhanced. The protective effect of GO supplementation helps mitigate oxidative stress in weaned piglets.

Higher villus height indicates good intestinal health, while lower villus height may be associated with intestinal inflammation, infection, or other digestive issues [22, 23]. Deeper crypts are generally associated with better barrier function and immune response, while shallower crypts may imply a compromised mucosal barrier or suppressed immune function [24]. Study have demonstrated that garlic gradually improves intestinal morphology in piglets or other species [13, 25, 26]. In this study, supplementation with 1 g/kg of GO led to an increase in jejunum villus height, with no notable effect on crypt depth. The variation observed can be attributed to the diverse responses of different parts of the intestine to growth promoters. It appears that distinct sections of the intestine exhibit varying reactions to exogenous growth promoters, although the underlying mechanisms remain unclear. Increased VH: CD are regarded as valuable indicators for assessing the nutrient digestion and absorption capacity of the small intestine [27]. Therefore, our findings suggest that GO can enhance digestion and absorption capacity in the jejunum, improving jejunum morphology.

LPS stimulates the immune system, triggering an inflammatory response that disrupts the balance between pro-inflammatory and anti-inflammatory cytokines in the gut, thereby exacerbating intestinal inflammation [28]. Supplementation with GO can prevent the LPS-induced increase in serum levels of IL-1β, TNF-α, and intestinal NFκB protein abundance. Additionally, intestinal barrier function is crucial for maintaining immune homeostasis in the gut. Epithelial damage due to bacterial antigen translocation into the lamina propria can lead to abnormal inflammation. Impairment of intestinal barrier function or increased intestinal permeability allows LPS and antigens to translocate into the subepithelial tissues, potentially enhancing the uptake of gut antigens and leading to mucosal oxidative stress and systemic inflammation [29, 30]. LPS disrupts normal mitochondrial function, leading to the release of free radicals, which induces lipid peroxidation, affecting membrane integrity and redox signaling, resulting in increased ROS and MDA levels and decreased glutathione activity [31, 32]. Compared to the LPS group, GO improved mitochondrial membrane potential, reduced ROS levels. However, despite the significant reduction in mitochondrial ROS production in the LPS + GO group, ROS levels remained relatively high compared to the CON group, indicating that GO supplementation only partially enhanced the antioxidant capacity of weaned piglets. Moreover, GO alleviated LPS-induced gut microbial dysbiosis by reshaping the microbiota composition. Notably, the relative abundance of the beneficial bacterium *Lactobacillus* was significantly increased, while the abundance of the pathogenic *Escherichia coli* was markedly reduced, suggesting a potential role of GO in restoring microbial homeostasis. In conclusion, the findings suggest that GO intervention improves intestinal barrier function and protects gut health by alleviating oxidative stress, inhibiting the excessive production of pro-inflammatory cytokines and restoring microbial homeostasis.

## Conclusion

In conclusion, supplementing the diet of weaned piglets with garlic essential oil has shown significant benefits. This 1 g/kg garlic essential oil has been demonstrated to boost growth performance, as evidenced by higher average daily weight gain and a more favorable feed conversion efficiency. Moreover, it reduces diarrhea incidence rates, promoting intestinal health. It positively affects the intestinal barrier supporting intestinal integrity and immune homeostasis. After LPS treatment, the preventive effects of 1 g/kg garlic essential and therapeutic effect of cefalexn on diarrhea were comparable. However garlic essential oil is more favorable to maintaining the intestinal microbiota homeostasis. Overall, these findings highlight garlic essential’s potential as a valuable dietary supplement for weaned piglets, deserving further investigation in animal husbandry practices.

## Supplementary Information

Below is the link to the electronic supplementary material.

Supplementary Material 1

Supplementary Material 2

## Data Availability

No datasets were generated or analysed during the current study.

## References

[1] Tang X, Xiong K, Fang R, Li M. Weaning stress and intestinal health of piglets: A review. Front Immunol. 2022;13:1042778. 36505434 10.3389/fimmu.2022.1042778 PMC9730250

[2] Millet S, Maertens L. The European ban on antibiotic growth promoters in animal feed: from challenges to opportunities. Vet J. 2011;187:143–4. 20627781 10.1016/j.tvjl.2010.05.001

[3] McKenzie P, Carter R. Change management reduces antibiotic use on pig farms. Aust Vet J. 2019;97:233–4. 31236927 10.1111/avj.12832

[4] Lillehoj H, Liu Y, Calsamiglia S, Fernandez-Miyakawa ME, Chi F, Cravens RL, Oh S, Gay CG. Phytochemicals as antibiotic alternatives to promote growth and enhance host health. Vet Res. 2018;49:76. 30060764 10.1186/s13567-018-0562-6 PMC6066919

[5] Borlinghaus J, Albrecht F, Gruhlke MC, Nwachukwu ID, Slusarenko AJ. Allicin: chemistry and biological properties. Molecules. 2014;19:12591–618. 25153873 10.3390/molecules190812591 PMC6271412

[6] Shi X, Zhou X, Chu X, Wang J, Xie B, Ge J, Guo Y, Li X, Yang G. Allicin Improves Metabolism in High-Fat Diet-Induced Obese Mice by Modulating the Gut Microbiota. *Nutrients* 2019, 11. 10.3390/nu11122909 PMC6949904 31810206

[7] Huang RH, Qiu XS, Shi FX, Hughes CL, Lu ZF, Zhu WY. Effects of dietary allicin on health and growth performance of weanling piglets and reduction in attractiveness of faeces to flies. Animal. 2011;5:304–11. 22440775 10.1017/S1751731110001953

[8] Cao S, Wang C, Yan J, Li X, Wen J, Hu C. Curcumin ameliorates oxidative stress-induced intestinal barrier injury and mitochondrial damage by promoting parkin dependent mitophagy through AMPK-TFEB signal pathway. Free Radic Biol Med. 2020;147:8–22. 31816386 10.1016/j.freeradbiomed.2019.12.004

[9] Huang L, Wan J, Chen Y, Wang Z, Hui L, Li Y, Xu D, Zhou W. Inhibitory effects of p38 inhibitor against mitochondrial dysfunction in the early brain injury after subarachnoid hemorrhage in mice. Brain Res. 2013;1517:133–40. 23603413 10.1016/j.brainres.2013.04.010

[10] Cao S, Wu H, Wang C, Zhang Q, Jiao L, Lin F, Hu CH. Diquat-induced oxidative stress increases intestinal permeability, impairs mitochondrial function, and triggers mitophagy in piglets. J Anim Sci. 2018;96:1795–805. 29562342 10.1093/jas/sky104 PMC6140957

[11] Cao S, Shen Z, Wang C, Zhang Q, Hong Q, He Y, Hu C. Resveratrol improves intestinal barrier function, alleviates mitochondrial dysfunction and induces mitophagy in Diquat challenged piglets(1). Food Funct. 2019;10:344–54. 30601541 10.1039/c8fo02091d

[12] Kairalla MA, Alshelmani MI, Aburas AA. Effect of diet supplemented with graded levels of Garlic (Allium sativum L.) powder on growth performance, carcass characteristics, blood hematology, and biochemistry of broilers. Open Vet J. 2022;12:595–601. 36589396 10.5455/OVJ.2022.v12.i5.1 PMC9789753

[13] Tatara MR, Sliwa E, Dudek K, Gawron A, Piersiak T, Dobrowolski P, Mosiewicz J, Siwicki A, Studzinski T. Aged Garlic extract and allicin improve performance and Gastrointestinal tract development of piglets reared in artificial Sow. Ann Agric Environ Med. 2008;15:63–9. 18581981

[14] Ayrle H, Nathues H, Bieber A, Durrer M, Quander N, Mevissen M, Walkenhorst M. Placebo-controlled study on the effects of oral administration of allium sativum L in postweaning piglets. Vet Rec. 2019;184:316. 30777882 10.1136/vr.105131

[15] Yan L, Kim IH. Effects of dietary supplementation of fermented Garlic powder on growth performance, apparent total tract digestibility, blood characteristics and faecal microbial concentration in weanling pigs. J Anim Physiol Anim Nutr (Berl). 2013;97:457–64. 22409599 10.1111/j.1439-0396.2012.01286.x

[16] Grela ER, Klebaniuk R. Chemical composition of Garlic Preparation and its utilization in piglet diets. Medycyna Weterynaryjna. 2007;63:792–5.

[17] Lan RX, Park JW, Lee DW, Kim IH. Effects of astragalus membranaceus, Codonopsis pilosula and allicin mixture on growth performance, nutrient digestibility, faecal microbial shedding, immune response and meat quality in finishing pigs. J Anim Physiol Anim Nutr (Berl). 2017;101:1122–9. 27868250 10.1111/jpn.12625

[18] Huang W, Yao C, Liu Y, Xu N, Yin Z, Xu W, Miao Y, Mai K, Ai Q. Dietary allicin improved the survival and growth of large yellow croaker (Larimichthys crocea) larvae via promoting intestinal development, alleviating inflammation and enhancing appetite. Front Physiol. 2020;11:587674. 33162901 10.3389/fphys.2020.587674 PMC7583326

[19] Chen YJ, Kim IH, Cho JH, Yoo JS, Wang Q, Wang Y, Huang Y. Evaluation of dietary l-carnitine or Garlic powder on growth performance, dry matter and nitrogen digestibilities, blood profiles and meat quality in finishing pigs. Anim Feed Sci Technol. 2008;141:141–52.

[20] Ziamajidi N, Nasiri A, Abbasalipourkabir R, Sadeghi Moheb S. Effects of Garlic extract on TNF-alpha expression and oxidative stress status in the kidneys of rats with STZ + nicotinamide-induced diabetes. Pharm Biol. 2017;55:526–31. 27937047 10.1080/13880209.2016.1255978 PMC6130558

[21] Askari M, Mozaffari H, Darooghegi Mofrad M, Jafari A, Surkan PJ, Amini MR, Azadbakht L. Effects of Garlic supplementation on oxidative stress and antioxidative capacity biomarkers: A systematic review and meta-analysis of randomized controlled trials. Phytother Res. 2021;35:3032–45. 33484037 10.1002/ptr.7021

[22] Savilahti E. Food-induced malabsorption syndromes. J Pediatr Gastroenterol Nutr. 2000;30(Suppl):S61–6. 10634301 10.1097/00005176-200001001-00010

[23] Tappenden KA. Intestinal adaptation following resection. Jpen J Parenter Enter Nutr. 2014;38:S23–31. 10.1177/0148607114525210 24586019

[24] Rao JN, Wang JY. Regulation of Gastrointestinal mucosal growth. Morgan & Claypool Life Sciences: San Rafael (CA); 2010. 21634069

[25] Liu X, Zhang X, Liu H, Fu H, Liu Y, Ge Y, Deng S, Tang Z, Mei L, Wang J, et al. Garlic-Derived Exosome-Like nanoparticles enhance gut homeostasis in stressed piglets: involvement of Lactobacillus reuteri modulation and Indole-3-propionic acid induction. J Agric Food Chem. 2025;73:7228–43. 40082245 10.1021/acs.jafc.4c11506

[26] Serrano-Jara D, Rivera-Gomis J, Tornel JA, Bernabe A, Martinez-Conesa C, Navarro JA, Canovas R, Otal J, Cubero MJ. Effects of dietary supplementation with purple Garlic powder and oregano essential oil on intestinal health in post-weaning piglets from commercial farms. Vet Res Commun. 2023;47:901–9. 36520356 10.1007/s11259-022-10053-2 PMC10209285

[27] Montagne L, Pluske JR, Hampson DJ. A review of interactions between dietary fibre and the intestinal mucosa, and their consequences on digestive health in young non-ruminant animals. Anim Feed Sci Technol. 2003;108:95–117.

[28] Di Vincenzo F, Del GA, Petito V, Lopetuso LR, Scaldaferri F. Gut microbiota, intestinal permeability, and systemic inflammation: a narrative review. Intern Emerg Med. 2024;19:275–93. 37505311 10.1007/s11739-023-03374-w PMC10954893

[29] Tilg H, Zmora N, Adolph TE, Elinav E. The intestinal microbiota fuelling metabolic inflammation. Nat Rev Immunol. 2020;20:40–54. 31388093 10.1038/s41577-019-0198-4

[30] Martel J, Chang SH, Ko YF, Hwang TL, Young JD, Ojcius DM. Gut barrier disruption and chronic disease. Trends Endocrinol Metab. 2022;33:247–65. 35151560 10.1016/j.tem.2022.01.002

[31] Tur J, Pereira-Lopes S, Vico T, Marin EA, Munoz JP, Hernandez-Alvarez M, Cardona PJ, Zorzano A, Lloberas J, Celada A. Mitofusin 2 in macrophages links mitochondrial ROS production, cytokine release, phagocytosis, autophagy, and bactericidal activity. Cell Rep. 2020;32:108079. 32846136 10.1016/j.celrep.2020.108079

[32] Su L, Zhang J, Gomez H, Kellum JA, Peng Z. Mitochondria ROS and mitophagy in acute kidney injury. Autophagy. 2023;19:401–14. 35678504 10.1080/15548627.2022.2084862 PMC9851232

