# Supplementary material for: Garlic essential oil alleviate oxidative stress, inflammation and microbiota dybiosis from small intestinal damage in lipopolysaccharide-challenged weaned piglets
Source: Porcine Health Manag. 2025 Sep 30;11:48. doi: 10.1186/s40813-025-00461-6 (PMC12486787; doi:10.1186/s40813-025-00461-6)
Supplement: Supplementary file 1 — Supplementary Material 1 [file 40813_2025_461_MOESM1_ESM.docx]

**Table S1 Main chemical constituents of garlic oil**

| Compound name | Molecular formula | Molecular weight (g/mol) | Retention time range (min) | | Relative content (%) | |
| --- | --- | --- | --- | --- | --- | --- |
|  |  |  | garlic oil | microcapsule | garlic oil | microcapsule |
| Diallyl disulfide | C_6_H_10_S_2_ | 146.28 | 11.631 | 11.424 | 42.61 | 41.92 |
| Diallyl trisulfide | C_6_H_10_S_3_ | 178.33 | 16.244 | 15.978 | 33.11 | 32.58 |
| Diallyl sulfide | C_6_H_10_S | 114.21 | 5.719 | 5.451 | 14.28 | 13.98 |
| Methyl allyl disulfide | C_4_H_8_S_2_ | 120.23 | 8.162 | 7.982 | 4.12 | 3.86 |
| Methyl allyl trisulfide | C_4_H_8_S_3_ | 152.28 | 13.268 | 13.117 | 3.18 | 2.77 |
| Tetraallyl disulfide | C_12_H_20_S_2_ | 232.41 | 18.511 | 18.072 | 1.25 | 0.95 |

The primary volatile sulfur compounds in garlic essential oil with relative contents above 1%. Garlic essential oil was extracted by steam distillation and analyzed using reverse-phase high-performance liquid chromatography with a C18 column (250 mm × 4.6 mm, 5 μm) under a methanol-water gradient elution. The flow rate was 1.0 mL/min, and the detection wavelength was 254 nm. Relative content percentages were calculated using the peak area normalization method and confirmed with standard compounds. Values represent averages from triplicate experiments.

**Table S2 Composition and nutrient levels of the basal diets**

| Item |  |
| --- | --- |
| Ingredient, % |  |
| Corn | 57.90 |
| Soybean meal | 25.47 |
| Fish meal | 5.00 |
| Whey powder | 4.00 |
| Cream powder | 4.50 |
| Limestone | 0.30 |
| CaH_2_PO_4_ | 1.20 |
| Moldproofant | 0.10 |
| Acidulant | 0.30 |
| L-Lysine HCl | 0.25 |
| Choline chloride | 0.10 |
| DL-Methionine | 0.05 |
| NaCl | 0.30 |
| Trace mineral premix^[a](https://www.sciencedirect.com/science/article/pii/S1567576915002027?via=ihub#tf0005)^ | 0.50 |
| Vitamin premix^[b](https://www.sciencedirect.com/science/article/pii/S1567576915002027?via=ihub#tf0010)^ | 0.03 |
| Total | 100.00 |
|  |  |
| Nutrient levels (M.J./kg, %)^[c](https://www.sciencedirect.com/science/article/pii/S1567576915002027?via=ihub#tf0015)^ | 14.02 |
| Crude protein | 20.05 |
| Ca | 0.62 |
| P | 0.50 |
| Lys | 1.19 |
| Met | 0.36 |
| Thr | 0.78 |
| Trp | 0.20 |
| Met + Cys | 0.65 |

Premix provided the following per kg of complete diet: Cu 15.5 mg, Fe 100 mg, Mn 30 mg, Zn 100 mg, Se 0.2 mg, and I 0.2 mg. VA 10,000 IU, VD3 1500 IU, VE 15 I.U., VK 0.5 mg, folic acid 0.2 mg, nicotinic 14 mg, pantothenate 10 mg, biotin 2.5 mg, V.B. 3 mg, VB24.0 mg, and VB12 0.01 mg.

**Table S3 QPCR primers**

| Gene^1^ | Primer squence（5’-3’） | Product（bp） | Annealing temperature（℃） |
| --- | --- | --- | --- |
| *IL-1β* | F: TGCCAGCTATGAGCCACTTCC | 130 | 56 |
|  | R: TGACGGGTCTCGAATGATGCT |  |  |
| *IL-10* | AGTTGCCTTCTTGGGACTGA | 102 | 52 |
|  | ACTGGTCTGTTGTGGGTGGT |  |  |
| *GPx1* | F: AGGGAAGCCGAGAACCACTA | 113 | 56 |
|  | R: CCAGCCCCTCATTCTCTTTCT |  |  |
| *NFkB* | F: TTCCGTCCCTCTCATACACTG | 198 | 56 |
|  | R: TTGAGATCTGCCCAGGTGGTA |  |  |
| *PPARα* | F: CTCGTGCAGGTCATCAAGAA | 158 | 61 |
|  | R: CAGCCCTCTTCATCTCCAAG |  |  |
| *Nrf2* | F: CAGTGCTCCTATGCGTGAA | 109 | 53 |
|  | R: GCGGCTTGAATGTTTGTC |  |  |
| *ZO-1* | F: CTGAGGGAATTGGGCAGGAA | 105 | 60 |
|  | R: TCACCAAAGGACTCAGCAGG |  |  |
| *Claudin-1* | F: GCCACAGCAAGGTATGGTAAC | 140 | 62 |
|  | R: AGTAGGGCACCTCCCAGAAG |  |  |
| *MUC1* | F: GTGCCGCTGCCCACAACCTG | 141 | 61 |
|  | R: AGCCGGGTACCCCAGACCCA |  |  |
| *β-actin* | F: TGCGGGACATCAAGGAGAAG | 196 | 60 |
|  | R: AGTTGAAGGTGGTCTCGTGG |  |  |
