# Supplementary figures and images for: Garlic essential oil alleviate oxidative stress, inflammation and microbiota dybiosis from small intestinal damage in lipopolysaccharide-challenged weaned piglets
Source: Porcine Health Manag. 2025 Sep 30;11:48. doi: 10.1186/s40813-025-00461-6 (PMC12486787; doi:10.1186/s40813-025-00461-6)

NFκB uncropped Gel


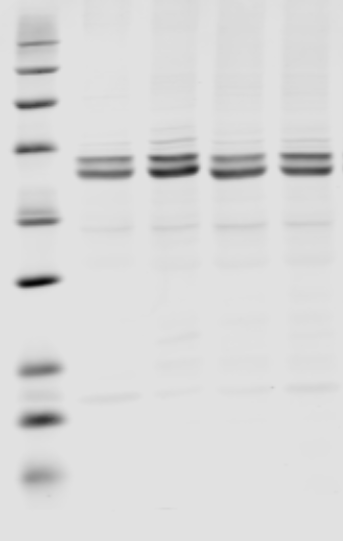


NFκB Blot


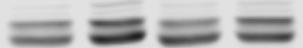


IL-10 uncropped Gel


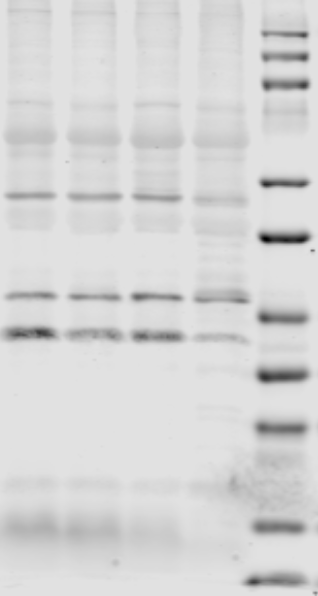


IL-10 Blot


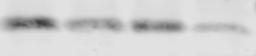

Supplement: Supplementary file 2 — Supplementary Material 2 [file 40813_2025_461_MOESM2_ESM.docx]
